# Platelet‐rich plasma intra‐articular knee injections from open preparation techniques do not pose a higher risk of joint infection: A systematic review of 91 randomized controlled trials and 5914 injections

**DOI:** 10.1002/jeo2.70002

**Published:** 2024-09-23

**Authors:** Mohammad Sami Alazzeh, Hamza Adnan Mohammad Naseh, Angelo Vasiliadis, Markus Laupheimer, Georgios Kalifis, Ayyoub Al‐Dolaymi, Luca Macchiarola, Theodorakys Marín Fermín

**Affiliations:** ^1^ Aspetar Orthopaedic and Sports Medicine Hospital Doha Qatar; ^2^ Department of Orthopaedic Surgery, Sports Trauma Unit St. Luke's Hospital Thessaloniki Greece; ^3^ Swisssportscare Zurich Switzerland; ^4^ The Centre for Sports & Exercise Medicine Queen Mary University of London London UK; ^5^ Thessaloniki Minimally Invasive Surgery (TheMIS) Orthopaedic Center St. Luke's Hospital Thessaloniki Greece; ^6^ Ospedale Casa Sollievo della Sofferenza San Giovanni Rotondo Foggia Italy; ^7^ Centro Médico Profesional Las Mercedes Caracas Venezuela

**Keywords:** closed technique, good manufacturing practices, joint infection, laminar flow, open technique, orthobiologics, platelet‐rich plasma

## Abstract

**Purpose:**

To compare the infection rate of intraarticular platelet‐rich plasma (PRP) knee injections between open and closed techniques in randomized controlled trials (RCTs) published in the last decade.

**Methods:**

Following the Preferred Reporting Items for Systematic Reviews and Meta‐Analyses guidelines, PubMed, Scopus and Virtual Health Library were accessed in October 2022 using the terms ‘platelet‐rich plasma’, ‘PRP’, ‘knee’ and ‘tibiofemoral’ alone and in combination with Boolean operators AND/OR. RCTs published during the last 10 years evaluating PRP intra‐articular knee injections were considered eligible. Studies were excluded if the kit/preparation technique was not described. Data were presented using individual studies' absolute values, totals, and pooled percentages. Publication bias was assessed using the ROBIS tool.

**Results:**

Ninety‐one studies met the predetermined eligibility criteria. Forty‐one implemented a closed technique, while 50 were open. All studies implementing a closed technique disclosed their commercial kits. Only 16 studies (17.58%) failed to report joint infections. Among the studies reporting joint infections as outcomes, 30 implemented a closed technique with 1195 patients, 1921 intra‐articular knee injections and 95.44% of patient follow‐up. On the other hand, 45 of them implemented an open technique with 2290 patients, 3993 intra‐articular knee injections and 97.07% of patient follow‐up. No patient had a joint infection among the included studies. Thirty‐three studies prepared their PRP in controlled environments (36.26%). Most studies did not report where the preparation occurred (48.35%). Only twelve studies disclosed using laminar flow during preparation (13.19%). The infection rate for both techniques was 0 per 1000 knee injections.

**Conclusion:**

Open PRP preparation techniques do not pose a higher risk of joint infection and can lower manufacturing costs when appropriate facilities are available. However, PRP preparation setting and laminar flow implementation data are deficient, and minimal requirements for good manufacturing practices demand further studies while adhering to local and regional regulations.

**Level of Evidence:**

Level I, systematic review of RCTs.

## Graphical abstract

Graphical summary of findings of a systematic review on the infection rate of intraarticular platelet‐rich plasma knee injections between open and closed techniques in randomized controlled trials published in the last decade.

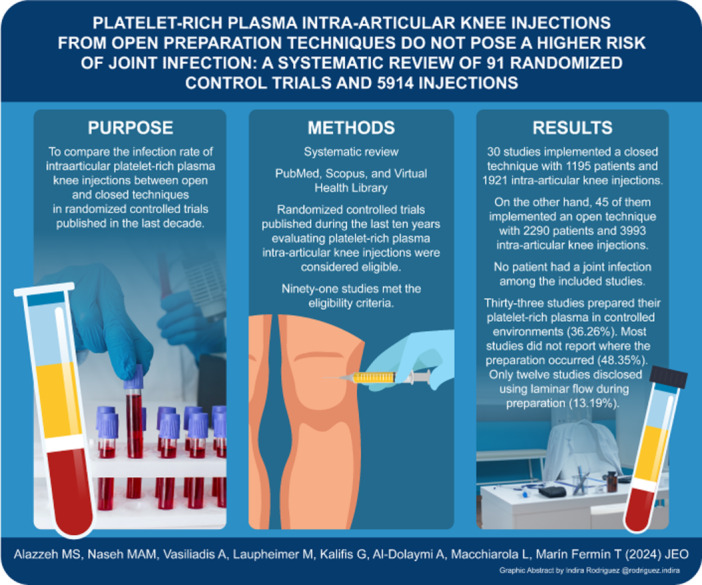

**Abbreviations**
PRP: platelet‐rich plasma
RCT: randomized controlled trial

## INTRODUCTION

Platelet‐rich plasma (PRP) is a portion of autologous blood with an increased platelet concentration obtained by centrifugation [72]. It is a widely implemented regenerative medicine therapy that aims to benefit healing maximally from platelet‐derived growth factors injected into the injury site [19]. Over the last two decades, its clinical implementation has thrived, including musculoskeletal pathologies such as knee osteoarthritis, patellar tendonitis and tennis elbow [37]. Patients are particularly interested in this treatment option driven by media, celebrity athletes, the desire for novel treatments and its autologous nature [3, 63].

PRP products and preparation methods are vast and explain the heterogeneous results reported in the literature [25, 64]. Preparation methods can be classified into open and closed techniques [2, 33, 39]. In open techniques, the plasma is exposed to the environment during its preparation in the working area through pipettes and collection tubes, potentially compromising its sterility. On the other hand, closed techniques allow blood sample processing and the resulting PRP without exposing it to the environment using commercial kits, yet significantly increasing its cost [7]. However, reports on joint infections from open PRP techniques are scarce. The present study aims to compare the infection rate of intraarticular PRP knee injections between open and closed techniques in randomized controlled trials (RCTs) published in the last decade.

## METHODS

This systematic review followed the Preferred Reporting Items for Systematic Reviews and Meta‐Analyses guidelines [67].

### Search strategy

Two independent reviewers identified Eligible articles through searches of PubMed, Scopus and Virtual Health Library databases up to October 31, 2022. The terms ‘platelet‐rich plasma’, ‘PRP’, ‘knee’ and ‘tibiofemoral’ were used alone and in combination with the Boolean operators AND and OR, and an RCT article‐type filter was applied.

### Eligibility criteria

Clinical studies evaluating PRP knee injections, regardless of the indication, were considered eligible for this systematic review if the following predefined criteria were fulfilled: (1) the study was conducted as an RCT; (2) PRP was given as a treatment in at least one arm of the study; (3) the study was published within the last 10 years and (4) the study was in English. Reference screening was performed on the potentially eligible articles, and an article was included when it met the eligibility criteria.

Studies were excluded if (1) PRP injections were not intra‐articular; or (2) the PRP kit/preparation technique was not described, or (3) plasma‐derived preparations other than PRP were implemented or (4) were reviews, abstracts, surveys, letters or editorials.

### Data extraction

Three investigators independently reviewed the included studies, and data were extracted to a predefined Excel spreadsheet with the following variables: (1) authors; (2) year of the study; (3) study; (4) PRP technique type (open vs. closed); (5) number of patients; (6) number of joint injections; (7) lost to follow‐up; (8) number of joint infections; (9) PRP preparation setting and (10) laminar flow implementation.

### Outcomes of interest

Open and closed PRP preparation techniques were defined according to Alves and Grimalt's [2]. Joint infections were accounted for when explicitly reported in the study. Studies with insufficient data to define the PRP preparation technique type or no explicit statement on the absence of joint infections were identified as ‘Not reported’ and excluded from the final analysis. The joint infection rate was defined as the number of joint infections per 1000 injections.

### Statistical analysis

Data were presented in tables using the individual studies' absolute values, totals, and pooled percentages derived from them. Statistical analysis was performed using SPSS V.19 and Microsoft Excel 2016 (Microsoft®, USA).

### Assessment of publication bias

The publication bias of the included studies was evaluated using the ROBIS tool [111]. This tool was rigorously developed to assess the risk of bias in systematic reviews, identify concerns with the review process and judge the risk of bias through signalling questions. These questions flag aspects of review design related to the potential for bias and aim to help assessors evaluate the risk of bias in the review process, results and conclusions.

## RESULTS

### Search results

**Figure 1:**
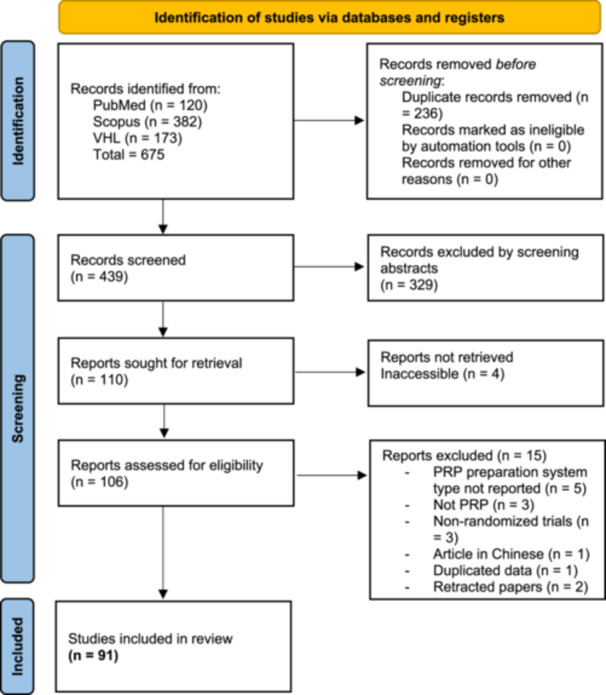
Flowchart of the literature search.

The initial literature search yielded 439 potentially relevant records after excluding duplicates. After screening titles and abstracts, 106 articles for full‐text evaluation were retrieved. Ninety‐one RCTs met the predetermined eligibility criteria [1, 4, 5, 6, 7, 8, 9, 10, 11, 12, 13, 14, 16, 18, 20, 21, 23, 24, 26, 27, 28, 29, 30, 31, 32, 34, 35, 36, 38, 40, 42, 43, 44, 45, 46, 47, 48, 49, 50, 52, 54, 55, 56, 58, 59, 60, 61, 66, 68, 73, 74, 75, 76, 77, 78, 79, 80, 81, 82, 83, 84, 85, 86, 87, 88, 89, 90, 91, 93, 94, 95, 96, 97, 98, 99, 100, 101, 102, 103, 104, 105, 106, 107, 108, 109, 113, 114, 115, 116, 117, 118, 119], and no additional study was included in the systematic review after citation screening (Figure 1).

### Synthesis of results

Forty‐one of the included studies implemented a closed PRP technique [1, 4, 5, 6, 8, 12, 14, 16, 20, 27, 28, 29, 32, 38, 42, 43, 44, 52, 54, 56, 58, 73, 74, 78, 79, 80, 81, 82, 84, 88, 96, 97, 98, 100, 103, 106, 107, 108, 113, 114, 119], while 50 were open [7, 9, 10, 11, 13, 18, 21, 23, 24, 26, 30, 31, 34, 35, 36, 40, 45, 46, 47, 48, 49, 50, 55, 59, 60, 61, 66, 68, 75, 76, 77, 83, 85, 86, 87, 89, 90, 91, 93, 94, 95, 99, 101, 102, 104, 105, 115, 116, 117, 118] (Table S1). All studies implementing a closed PRP technique disclosed their commercial kits. Only 16 studies (17.58%) failed to report joint infections among their outcomes [1, 5, 10, 14, 16, 18, 21, 24, 27, 38, 80, 82, 85, 88, 106, 114].

Among the studies reporting joint infections as outcomes, 30 implemented a closed PRP technique with 1195 patients, 1921 intra‐articular knee injections and 95.44% of patient follow‐up [4, 6, 8, 12, 20, 28, 29, 32, 42, 43, 44, 52, 54, 56, 58, 73, 74, 78, 79, 81, 84, 96, 97, 98, 100, 103, 107, 108, 113, 119]. On the other hand, 45 of them implemented an open PRP technique with 2290 patients, 3993 intra‐articular knee injections and 97.07% of patient follow‐up [7, 9, 11, 13, 23, 26, 30, 31, 34, 35, 36, 40, 45, 46, 47, 48, 49, 50, 55, 59, 60, 61, 66, 68, 75, 76, 77, 83, 86, 87, 89, 90, 91, 93, 94, 95, 99, 101, 102, 104, 105, 115, 116, 117, 118]. No patient had a joint infection among the included studies.

Thirty‐three studies prepared their PRP in controlled environments (36.26%). Twelve studies reported preparing the PRP in laboratories [1, 6, 11, 26, 30, 46, 78, 80, 81, 82, 105, 115], nine inside the operating room [21, 27, 44, 49, 56, 60, 66, 102, 106], eight in transfusional medicine services [23, 24, 34, 59, 68, 75, 118] and four in clean rooms [48, 61, 94, 99]. Most studies did not report where the PRP preparation occurred (48.35%). Only 12 studies disclosed using laminar flow during PRP preparation (13.19%) [9, 26, 36, 48, 61, 68, 75, 93, 94, 99, 105, 117]. The infection rate for both techniques was 0 per 1000 knee injections.

### Risk of publication bias results

**Figure 2:**
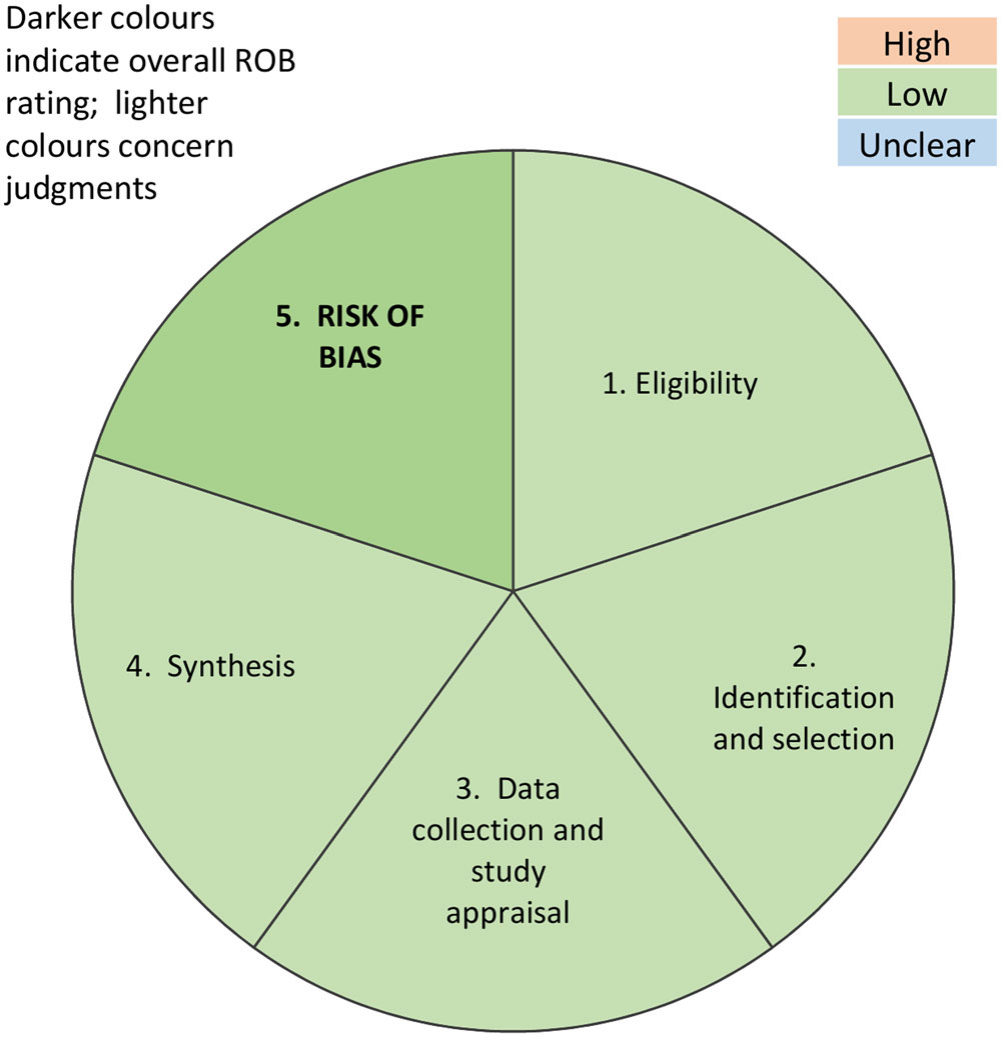
Assessment of publication bias with ROBIS tool.

The overall risk of bias was found to be low in all domains (Figure 2). However, missing information on PRP preparation setting among the studies may limit the extent of the study's conclusions.

## DISCUSSION

The main finding of the present systematic review is that open PRP preparation techniques do not pose a higher risk of joint infection. In fact, among 45 RCTs, 3993 intra‐articular knee injections and more than 95% follow‐up, no joint infection was reported. However, PRP preparation setting and laminar flow implementation data are deficient.

Current good manufacturing practices recommend that open techniques be handled in a Class A environment under laminar flow to diminish product contamination to negligible levels, restricting its implementation to specialized facilities [51, 92]. However, it may vary depending on local and regional regulations. The present study demonstrates that the joint infection rate after intra‐articular PRP knee injections prepared with open techniques is not higher than those prepared with closed ones. Nevertheless, the preparation setting and handling seem more relevant in preventing potential PRP product contamination than the technique. The deficient report on PRP preparation setting and laminar flow implementation among the included studies limits further recommendations.

The poor reporting quality among orthobiologics studies has been well‐documented [15, 22, 64, 71]. This study contributes to the existing literature and calls attention to journals and reviewers for the mandatory adherence to the available reporting guidelines in the submission and reviewing process [65]. Even more, current reporting guidelines fail to consider preparation settings among the relevant items that would provide scientific background in determining the minimum requirements to offer this therapy [53, 69, 70].

From an economic perspective, the cost reduction from using open PRP preparation techniques seems significant in centres with appropriate facilities [33, 68]. Available PRP commercial kits can range from US$300 to US$1.500 [3, 62], and knee injection costs can rise to US$2500 [33]. Furthermore, it has been reported that the charge data do not correlate with the cost of the goods and services required for PRP therapy [109]. Open techniques can reduce the cost of PRP to as low as US$42, which can be especially relevant in cost‐sensitive populations [7, 33, 112].

The present systematic review conveys the limitations of the analyzed studies. Conducting RCT studies demands the highest methodological quality and patient safety protocols. Thus, the joint infection rate derived from them might be lower than standard clinical practice and should not be extrapolated to office‐based settings. Cautious interpretation of the results of this study is encouraged, and meticulous and sterile PRP preparation to minimize the risk of infection when offering PRP therapy is recommended [17]. Finally, in vitro and animal studies on PRP have shown an intrinsic antimicrobial effect and minimal contamination rates [41, 57, 110]. This antimicrobial effect includes methicillin‐sensitive and methicillin‐resistant *Staphylococcus aureus* and Group A *Streptococcus* [41, 57]. Future studies should aim to assess the minimal PRP manufacturing requirements, including the need for laminar flow.

## CONCLUSION

Open PRP preparation techniques do not pose a higher risk of joint infection. Open PRP preparation techniques can lower manufacturing costs when appropriate facilities are available. PRP preparation setting and laminar flow implementation data are deficient, and minimal requirements for good manufacturing practices demand further studies while adhering to local and regional regulations.

## AUTHOR CONTRIBUTIONS

**Mohammad Sami Alazzeh**: Methodology; validation; investigation; resources; data curation; writing—review and editing. **Hamza Adnan Mohammad Naseh**: Methodology; validation; investigation; resources. **Angelo Vasiliadis**: Investigation; data curation; writing—review and editing. **Markus Laupheimer**: Supervision; project administration. **Georgios Kalifis**: Methodology; validation; investigation. **Ayyoub Al‐Dolaymi**: Methodology; validation; investigation. **Luca Macchiarola**: Methodology; validation; investigation. **Theodorakys Marín Fermín**: Conceptualization; methodology; validation; formal analysis; investigation; resources; data curation; writing—original draft; visualization; supervision; project administration.

## CONFLICT OF INTEREST STATEMENT

The authors declare no conflict of interest.

## ETHICS STATEMENT

No ethical approval was required for the presented study. No informed consent was required for the presented study.

## Supporting information

Supplementary Information

## Data Availability

The data underlying this article are available here and in Supporting Information.
